# Community participation in the health system: analyzing the implementation of community health committee policies in Kenya

**DOI:** 10.1017/S1463423623000208

**Published:** 2023-04-28

**Authors:** Robinson Karuga, Marjolein Dieleman, Patrick Mbindyo, Kim Ozano, Judy Wairiuko, Jacqueline E.W. Broerse, Maryse Kok

**Affiliations:** 1 LVCT Health, Nairobi, Kenya; 2 Athena Institute, Vrije University, Amsterdam, The Netherlands; 3 KIT Royal Tropical Institute, Amsterdam, Netherlands; 4 Jomo Kenyatta University of Agriculture and Technology, Nairobi, Kenya; 5 The SCL Agency, Five Fords Gate, Wrexham, Wales, UK; 6 Directorate of Preventive and Promotive Health, Nairobi City County, City Hall Way, Nairobi, Kenya

**Keywords:** Community health committees, community health services, community participation, decentralization, health policy

## Abstract

**Background:**

Community health committees (CHCs) are a mechanism for communities to voluntarily participate in making decisions and providing oversight of the delivery of community health services. For CHCs to succeed, governments need to implement policies that promote community participation. Our research aimed to analyze factors influencing the implementation of CHC-related policies in Kenya.

**Methods:**

Using a qualitative study design, we extracted data from policy documents and conducted 12 key informant interviews with health workers and health managers in two counties (rural and urban) and the national Ministry of Health. We applied content analysis for both the policy documents and interview transcripts and summarized the factors that influenced the implementation of CHC-related policies.

**Findings:**

Since the inception of the community health strategy, the roles of CHCs in community participation have been consistently vague. Primary health workers found the policy content related to CHCs challenging to translate into practice. They also had an inadequate understanding of the roles of CHCs, partly because policy content was not adequately disseminated at the primary healthcare level. It emerged that actors involved in organizing and providing community health services did not perceive CHCs as valuable mechanisms for community participation. County governments did not allocate funds to support CHC activities, and policies focused more on incentivizing community health volunteers (CHVs) who, unlike CHCs, provide health services at the household level. CHVs are incorporated in CHCs.

**Conclusion:**

Kenya’s community health policy inadvertently created role conflict and competition for resources and recognition between community health workers involved in service delivery and those involved in overseeing community health services. Community health policies and related bills need to clearly define the roles of CHCs. County governments can promote the implementation of CHC policies by including CHCs in the agenda during the annual review of performance in the health sector.

## Background

**Figure 1.**
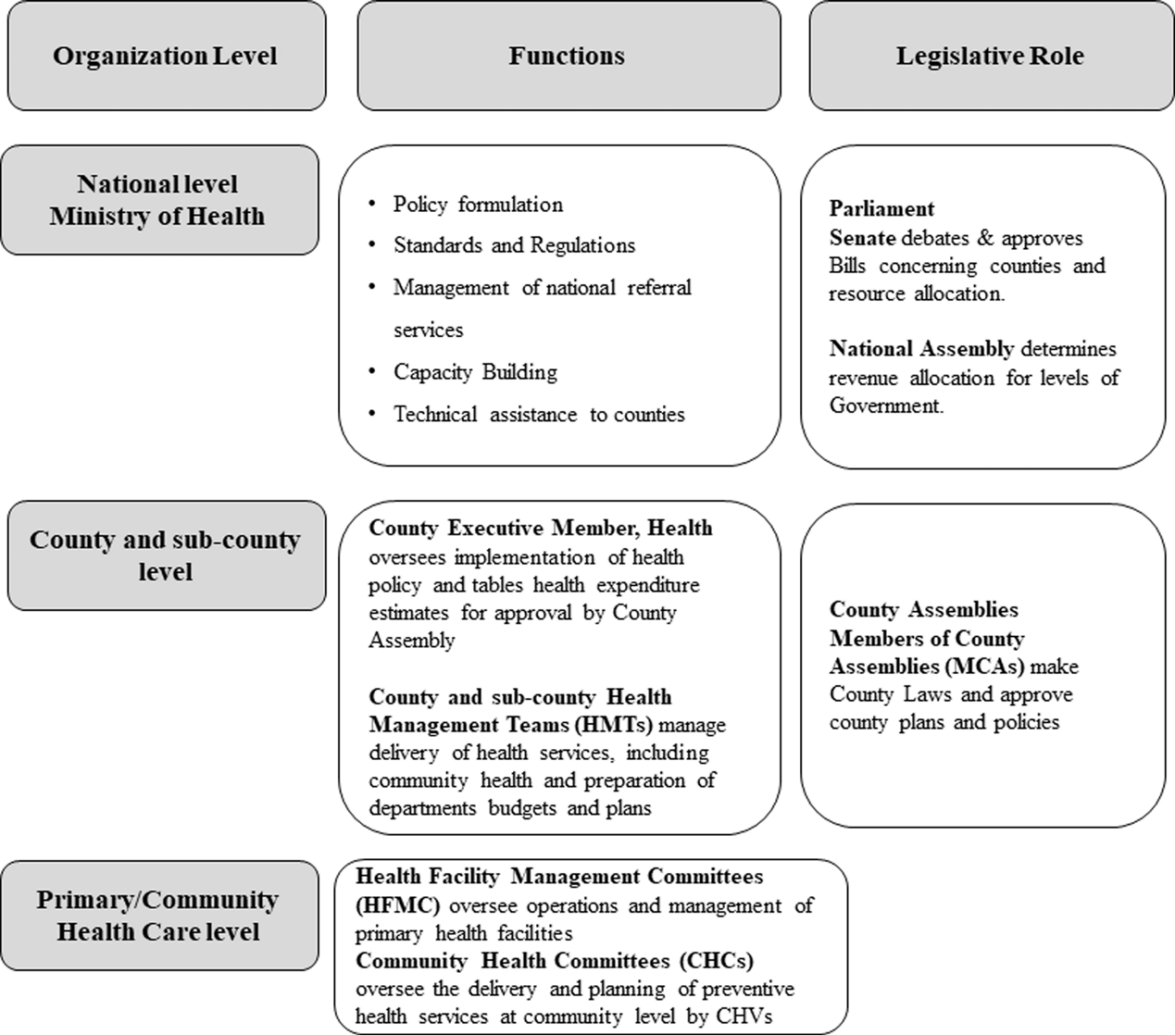
Illustration of Kenya’s devolved health system (Ministry of Health, 2014)

Since the early 1980s, policymakers have touted that the participation of communities in making decisions about their primary health care (PHC) services increases their control over local health priorities (Mehrotra and Jarrett, 2002). Among the several global and regional policies that aim to strengthen community participation in PHC, the Bamako Initiative specifically sought to empower communities to participate in the management of PHC in decentralized health systems (World Health Organization, 1987). For community participation to succeed, governments need clear commitments to adopt and implement policies that legitimize community-level governance structures (Jarrett and Ofosu-Amaah, 1992). Decentralization of health systems is an essential reform process that creates opportunities for community members to participate in the management of health resources (Bossert and Beauvais, 2002). Kenya devolved the management of PHC (including community health services) and primary referral hospitals to 47 semi-autonomous county governments after the 2013 general elections. The national Ministry of Health (MoH) maintained functions related to health policy formulation, regulation, and technical support, as summarized in Figure1 (Government of Kenya, 2010). Devolution of health services opened up the decision space for county governments to implement health policies in ways that are responsive to the local community’s needs and contexts (McCollum *et al*., 2018; Tsofa *et al*., 2017a; 2017b).

## Community health services in Kenya

Community health volunteers (CHVs) deliver preventive and basic curative care at the community and household levels under the supervision of salaried community health assistants (CHAs). Community members are supposed to participate in overseeing and making decisions on community health services through community health committees (CHCs). CHCs are supposed to enable community members to actively share information, consult with primary health workers on health matters, and make decisions about priority health interventions at the community level (Molyneux *et al*., 2012). CHCs are expected to (1) provide leadership and oversight in the implementation of community health services; (2) develop community health annual work plans that are incorporated into the local PHC plans; (3) coordinate community health dialogues and health action days; (4) mobilize resources for community health services; (5) promote social accountability in the delivery of PHC; (6) engage in essential human resource and financial management in the community; and (7) mobilize community members to participate in community health activities (Ministry of Health, 2014). CHC members are supposed to be elected by community members during community *barazas* (administrative meetings with community elders and community members) to allow for representation of different community groups (women, youth, persons with disabilities, etc.) in a community health unit, which is a geographical area in which community health services are delivered. According to the Community Health Policy, CHCs should have between five and seven members who serve a 3-year term that is renewable once, unless the community agrees to extend their term (Ministry of Health, 2020a). CHC members are required to elect a chairperson from the local community who is a community member. Each CHC is required to have a maximum of two CHVs. The chair of the CHC is a co-opted member of the local health facility management committee (Ministry of Health, 2014). A recent study found that community participation through CHCs seems constrained. Despite CHCs being made up of influential persons in their communities, they had little control over the flow of health-related information and were peripheral actors in community health networks. Most primary health workers were not aware of CHC roles, while others did not involve them in developing community health plans (Karuga *et al*., 2019a). This evidence points to a gap between policy intentions and the implementation of community health policy (Teddy *et al*., 2019, Hamra *et al*., 2020).

Implementation of health policies is a complex process that involves working with and through actors who are influenced by their understanding of the policy content and context (Gilson, 2016; Campos and Reich, 2019). This article aims to provide a deeper understanding of the policy-related factors that influence the implementation of CHC-related policies in a rural and urban settings in Kenya.

## Methods

### Study design

This qualitative study was conducted in two purposively sampled counties (urban and rural) where we had previously studied the contextual factors that influenced the performance of CHCs *(Submitted for publication*). The qualitative research approach allowed us to analyze both retrospective and current events in developing and implementing community health policies (World Health Organization, 2012, Fitzgerald, 1999). The rural and urban study counties are in western and central Kenya. The rural county’s predominant economic activities include micro-enterprise, fishing, agriculture, and small-scale mining. The metropolitan study county is highly cosmopolitan and characterized by wide socioeconomic and health status gaps. The majority of residents in the lower socioeconomic status live in informal settlements, and most are involved in either menial labor or running micro-enterprises.

### Data collection methods

Between April and June 2021, we concurrently conducted document analysis and key informant interviews. An initial document analysis helped us obtain content that informed our interview topic guides. The Walt & Gilson policy analysis framework also informed our data collection tools, i.e., we sought to discuss issues related to actors, content, context, and process to analyze any gaps in the implementation of CHC-related policies (Walt and Gilson, 1994).

#### Document analysis

We analyzed documents using the READ approach for health policy analysis (Supplementary File 1) (Dalglish *et al*., 2020). We searched for publicly accessible documents (guidelines, strategies, policy, and bills) from the Ministry of Health and county government websites published between 2006 (when the first national community health strategy was launched) and 2021 (when the most recent community health strategy was launched). We used search terms such as “Community Health Committee,” “Community Health Policy,” “Community Health Services Bill,” AND “[county names].” We requested additional and relevant documents from key informants during the interviews. We extensively read 21 policy documents and extracted data from 13 documents that addressed the implementation of community health policies and governance of community health services (Dalglish *et al*., 2020). While analyzing policy documents, we extracted data on policy objectives, policy frameworks, debates during legislative proceedings, and training content for CHCs. Table 1 summarizes the documents that we analyzed.

**Table 1.** Documents included in the analysis

Document category	Year of publication	Document title	Level of policy
**Operational documents** These were policies, Health Sector Strategies, official declarations	2020	Ministry of Health. Kenya Community Health Strategy 2020–2025	National
	2020	Ministry of Health. Kenya Community Health Policy 2020 – 2030	National
	2019	Ministry of Health. Kenya Primary Health Care Strategic Framework 2019–2024	National
	2019	Ministry of Health. Planning, Budgeting and Performance Review Process Guide for Health Sector: Simple Guide to MTEF for Health Sector. Nairobi, Kenya	National
	2014	Ministry of Health. Strategy for Community Health (2014-2019): Transforming Health: Accelerating Attainment of Health Goals	National
**Legal documents** included laws, Bills, and any cooperative agreements	2020	Kenya Senate. The Community Health Services Bill, 2020	National
	2019	[Urban] County Community Health Services Act 2019	County
	2019	Hansard Report Second [Urban] County Assembly – Third Session 18 Sept 2019	County
	2019	Hansard Report Second [Urban] County Assembly – Second Session 11 Sept 2019	County
	2020	[Rural] County Community Health Services Bill, 2020	County
	2012	Ministry of Health. Handbook for Community Health Committees.	National
	2006	Ministry of Health. Community Health Strategy	National
	2007	Community Strategy Implementation Guidelines For Managers of the Kenya Essential Package for Health at the Community Level	National

#### Key informant interviews

We conducted key informant interviews with 12 purposefully sampled key informants. These key informants were stakeholders who were actively involved in designing and implementing community health policies at either national or county levels. We sampled a combination of technical experts, decision-makers, and primary health workers that were part of Community Health Technical Working Groups at the national MoH and at county level (Gentles *et al*., 2015; Palinkas *et al*., 2015; Palys, 2008). Key informants that consented to participate included two national-level managers, two county health managers, three sub-county health managers, and five primary health workers. The interviews explored our key informants’ contribution and experiences during the policy implementation process, their views on the actors that influenced the implementation of community health policies; how these policies influenced CHCs and how contextual factors (e.g., political, socio-economic, administrative, etc.) influenced the implementation of policies related to CHCs. Since we conducted this study during the COVID-19 pandemic, we opted to conduct interviews over the phone and using virtual meeting platforms. We invited our key informants for interviews via email or telephone after obtaining administrative clearance from their supervisors. We then shared consent forms and study information sheets with key informants in advance using email or WhatsApp© for participants in remote areas who had unreliable internet access. We asked participants if they could return signed consent forms before the interviews. Those who could not append electronic signatures consented verbally before the interviews began. Phone and virtual interviews with key informants lasted between 20 and 47 min. The narratives from key informant interviews and content from the document analysis enabled us to reach data saturation, where no new information emerged from both interviews and the document analysis (Guest *et al*., 2020; Gentles *et al*., 2015).

#### Data analysis

A team of four researchers transcribed the digital audio recordings in MS Word© and re-read them to ensure that they accurately captured the interview discussions. These transcripts were then uploaded into Nvivo R1 (QSR International, Australia) analysis software for coding and charting. We applied the content analysis approach to analyze and interpreted the contents from the document analysis and interviews. During analysis, the primary author (RK) and two research assistants re-read the transcripts to inductively identify key themes that emerged from the transcripts and developed an initial coding framework. Inter-coder reliability between the three researchers was tested by piloting the initial coding framework with three randomly selected transcripts. We then resolved any discrepancies in coding and finalized the coding framework. Once agreement on the final coding framework was reached, we coded data from policy documents and interview transcripts into the themes that best described the policy-related factors that influenced CHCs based on our data (Hsieh and Shannon, 2005; Elo and Kyngäs, 2008). We then summarized narratives from the transcripts and document analysis by the emerging main themes and sub-themes.

## Results

The four main themes that emerged from our data were: (1) evolving policy content on CHCs, (2) inadequate clarity on policy content relating to CHCs, (3) the perceived value of CHCs among actors at national and sub-national levels, and (4) legal frameworks on CHCs.

1. **Evolving policy content on CHCs**

Our document analysis found that policy content on CHCs has been evolving since the first community health strategy was launched. This evolution has had implications for the operations of CHCs. The first community health strategy that was launched in June 2006 focused on defining community-level health services and strengthening the link between community members and PHC facilities. This policy was vague on how community members would participate in management, planning, and decision-making in community health. For example, district-level health management teams (DHMTs) were responsible for mobilizing financial support for the community health strategy from political leaders, religious leaders, and community-based organizations (CBOs). The initial community health strategy briefly mentioned that village health committees (VHCs) would be responsible for overseeing community health services. Still, VHCs were not factored into the implementation framework as a specific component of community health services. The 2006 community health strategy identified nongovernmental organizations (NGOs), CBOs, Community Health Extension Workers *(Now referred to as Community Health Assistants, CHAs)*, and Community Owned Resource Persons (CORPs) *(now called CHVs)* as key actors in the establishment of community health units. VHCs were not listed as actors in the establishment of community health units. The strategy stipulated that VHCs should report to the Chair of the Dispensary Management Committee on matters related to community health services.

*“Through the committee* [VHC], *the Chair will mobilize community resources and undertake social mobilization for implementation, reporting to the Dispensary Committees”* **(Community Health Strategy, 2006, page 24)**

In March 2007, the MoH released a guideline for implementing community health services. This implementation guideline introduced CHCs as governance structures responsible for overseeing the delivery of community health services. This guideline further provided details on the composition of CHCs and their roles (pg. 6). However, the role of CHCs in establishing community health units and the selection of CHVs remained vague. Furthermore, the guideline clearly defined the training content for CHVs but did not address the training of CHCs. Performance indicators listed in this guideline focused on community health service delivery by CHVs, and there were no performance indicators for CHCs.

The next strategy for community health services was launched in 2014, as county governments were taking over devolved health functions. The roles of CHCs stipulated in this strategy were broad and vague (pg 19). For the first time, this strategy introduced the need for developing legal frameworks that would allow for CHCs to be recognized as legitimately constituted community-level governance structures in the health system. The third national Community Health Strategy (2020-2025) pointed out weaknesses in community health services identified through a nationwide survey (Health, 2019). In response to the evidence from the survey, this national strategy presents more apparent roles and responsibilities of CHCs compared to earlier strategies. It emphasizes the importance of strengthening governance in community health. This strategy spells out clear intentions to

*“review, redesign, and dissemination of community health committee (CHC) guidelines, reviewing training manual for CHCs and involvement of sub-national level Health Management Teams in the reconstitution of CHCs for ownership of all community-led health activities”* [**page 15**].

This strategy lays out clear advocacy plans, timelines, and budgets for developing legal frameworks for CHCs at the national and county levels. This community health strategy also contains detailed plans for how counties can monitor the performance of CHCs through the development of accountability mechanisms to assess the functionality of CHCs, conducting bi-annual meetings to review resource allocation and utilization, and quarterly reviews of CHC performance. According to the third national community health strategy, all 47 counties in Kenya are expected to have trained CHCs in all community health units and have systems in place for monitoring the performance of CHCs by 2025. The role of CHCs, thus, became more prominent with the evolution of policy content in Kenya’s Community Health Strategy.

1. **Inadequate clarity of CHC policy content**

Our interviews with health managers and primary health workers revealed that policies on CHCs were not adequately clear to actors that were responsible for implementing them. Lack of clarity about CHC-related policies was associated with an inadequate understanding of CHC roles among community health actors – rural and urban settings – and complex content in CHC training materials.

### Inadequate understanding of CHC roles among actors involved in implementing policies

After formulation or revising health policies, the national MoH disseminates these policy updates to selected county-level officials, who are then tasked with further dissemination and sensitization of managers and health workers in their respective counties, with support from their county governments. From our interviews, we found that over time, only some health workers and managers received updates on new developments in community health policies, such as the community health strategy that is updated every 5 years. Primary health workers in the urban county reported receiving updated community health strategies via social media platforms such as WhatsApp ©. Primary health workers in the urban county reported taking personal initiative to apprise themselves on community health policies and implement these policies as they interpreted them, as illustrated in this response to a question on how they were sensitized to CHC policies:

*“Mostly, I can say WhatsApp© groups for our* [professional] *association… The coordinator or whoever shares the policy. It’s not done officially. You are supposed to download it. It’s for you to pick it or leave it…. So it is upon you as an individual may be to download the policy, go through it by yourself, understand it your way, interpret your way, work within the way you want.”* **(Male Primary Health Worker, Urban County)**

All five participants from the rural county were not aware that the MoH had released the 2020–2025 Community Health Strategy. They were also not aware that there was a CHC training handbook of 2012, and that this training handbook was being reviewed by the MoH, at the time of this study. As a result of lacking awareness, primary health workers either did not implement CHC policies or they implemented CHC policies based on their interpretations on how community participation should be implemented, as one primary health worker remarked when she was asked if she was aware of the updated Community Health Strategy and the CHC training manual during an interview:

*“Okay, for me, I still take it as a rumor* [referring to the updated Strategy] *because it’s not something that has been rolled down to us, you know, you can only defend what you know, but now, we still treat it as a rumor but when it will be implemented, is when I can talk boldly about it”* **(Female Primary Health Worker, Rural County)**

We noted from our interviews that primary health workers, especially in the urban county, required guidance on contextualizing the implementation of policies on CHCs. Primary health workers reported that policy content on managing CHCs was vague. As a result, they implemented CHC policies based on their interpretation and in ways that made their work easier, especially in the formation of CHCs and managing community participation, as presented in this interview excerpt:

*“…it doesn’t make sense, whereby we have 10 CHVs in one* [community] *unit, and you have ten or more CHC members. There’s a gray area that needs policy guidance….It’s not realistic to put it* [implement the policy] *in context. So, we are combining two or three, or four community units to be governed by one CHC. Because I mean, you know, that makes our work easier. It is like our modus operandi* [laughs].” **(Male Primary Health Worker, Urban County)**

### Complex CHC training content

The primary document for training CHCs is the CHC Training Handbook, published by the national MoH in 2012, with technical and financial support from NGOs and a bilateral donor. The MoH initiated the process of updating this Training Handbook in 2021. Besides providing details on the formation, roles, and competencies of CHC members, training content in the CHC Training Handbook is organized into seven modules, to be covered within seven days. The modules in this handbook are leadership in community health, governance and community health services, roles of CHC in effective communication, advocacy, networking, and social mobilization in the community unit, personnel management issues, resource mobilization, proposal writing, and financial management, community health information system, and monitoring and evaluation. The training content is mainly delivered using lectures that range between 1 and 2 hours long, with each day of training having about 7 hours of lecture time.

The training plan and approaches in the CHC Training Handbook do not provide room for critical reflection for CHC participants and do not have vignettes and worked examples for modules such as proposal development, resource mobilization, financial management, and community health information system. The training approach in this training manual does not consider that most CHC members may be advanced in age and may have limited education. Most of the training content was taken from advanced professional management-level training resources such as textbooks and websites, and the language on principles and theories of management and leadership is quite complex. Here is an excerpt from a session on leadership in the CHC Training Handbook:

*What motivates employees to go to work each morning? Social psychologist Douglas McGregor of the Massachusetts Institute of Technology expounded on two contrasting theories on human motivation and management in the 1960s: The X Theory and the Y Theory.*

- *Comparison of X and Y leadership*
- *Application of X and Y styles*

*(Page 34 and 35)*

The complexity of the CHC training manual was confirmed by three interview participants, as shown in this excerpt by a community health services manager:

*“It was too technical* [training content], *and people felt that it left out some vital issues like that one on data, yeah. Some things were missing…*” **(Female Health Manager, Urban County)**

1. **Perceived value of CHCs among government and non-government health system actors**

Our interviews revealed that county health managers did not perceive CHCs as important community health governance structures. This was revealed by data on the development and implementation of CHC policies. Most participants that we interviewed had the perception that actors (politicians, health managers, and NGO staff) who were involved in the development of community health policies at both national and county levels considered CHCs less critical compared to CHVs and as such CHCs did not have an active role in the delivery of community health services. Our interviewees reported that policy actors prioritized developing national and county-level community health policies aimed at motivating and retaining CHVs, as seen in this interview excerpt with a primary health worker:

*“When he* [Chair of a Professional Association] *tried raising the point of CHCs and CHVs, he was pinned down. He was told, “we are supporting people who are going directly to households, not people who don’t have a specific role directly in the household,” from the county team…. So now they said these volunteers providing services at the household level are the people with more significance and more relevance. That was also the argument of the Members of the County Assembly in the health committee”* **(Male Primary Health Worker, Urban County)**

Analysis of minutes of legislative proceedings in the urban County Legislative Assembly revealed that Members of County Assemblies (MCAs - county-level legislators) placed more value on the roles of CHVs in community health services. During the debate on the urban county community health services bill, MCAs debated the importance of remunerating CHVs and providing them with medical insurance cover. During one debate session, only one legislator mentioned governance structures in community health service delivery.

Our interview participants pointed out that County Health Management Teams (CHMTs) did not allocate financial resources for supporting CHC activities such as formation processes, training CHCs, providing allowances, and supporting community participation events. One national-level interview participant reported that some County Health Departments received funds from donor agencies for training CHCs on their roles in governance. Still, county managers opted to utilize these resources to train their CHMTs and health facility management teams instead, as seen in this excerpt:

*“So in all the counties when this support* [financial support for training CHCs from a donor] *was taken there, the counties said that we can’t use these resources to train CHCs. We want to use these resources to train to train CHMTs and facility management committees. So, that would mean that these counties do not understand the role of CHCs in managing health services”*. **(Male National Level Health Manager)**

We observed a perceived low value of CHCs in the health system among different actors during the interviews. Primary health workers that we interviewed from both the rural and urban counties reported that they were not held accountable, whether or not they engaged CHCs in the implementation of community health services.

*“You know, for us, as long as you have engaged the CHVs, no one cares if you have involved the CHCs or not. We have targets for quarterly dialogue meetings and monthly action days. These are the targets we have per community unit. Nobody comes to scrutinize the quality of how it is done. So long as you list whom you did it with, it doesn’t matter, as long as you have reported.”* **(Male Primary Health Worker, Urban County)**

Our interview participants narrated that county governments allocated funds to support governance activities in PHC facilities (health centers and dispensaries) and not for community health services through CHCs. County managers perceived Health Facility Management Committees (HFMCs), which oversee services in primary health facilities, as more critical governance structures compared to CHCs because they are responsible for funds disbursed from counties to these primary health facilities. Three interview participants noted that in their contexts, it may be beneficial to embed CHCs as part of the facility management committees, where they can advocate for resources to oversee the implementation of community health services.

*“I think they* [CHC] *can be anchored in the health facility management committee. You know, in the health facility management committee, one of them is a CHC member, and so this CHC member can give a recommendation that because all these community units fit in the health facility, maybe they can get a sitting allowance, or a budget for what they do. Yeah, but it’s a bit tricky you know.”* **(Female Health Manager, Urban County)**

Our interviewees reported that due to the limited funding allocated for the implementation of community health services, national and subnational-level health managers relied on external donors and non-government organizations (NGOs) to finance the implementation of community health policies, which also influenced the roles of CHCs. Our interview participants reported that donors and NGOs, who primarily financed community health services, preferred working with CHVs and not CHCs. According to our interviewees, NGO actors perceived the engagement of CHCs while implementing community health programs as a “delay” in time-sensitive donor-funded projects. Dependence on NGOs for financing community health services meant that primary health workers further neglected the role of CHCs in overseeing these community-level interventions as observed by one health manager:

*“When partners* [NGOs] *are budgeting for* [community health] *activities, they don’t budget for the CHCs because I think even the donor world wants to see performance-based results. So, they value CHVs because they are the ones who will make them achieve their maternal and child health indicators as opposed to a CHC.”* **(Female Manager, Urban County)**

1. **Legal frameworks on CHCs at national and county levels**

In September 2019, members of the County Legislative Assembly in the urban study county approved the Community Health Services Act, which provided a legal framework for the formation and composition of CHCs. This county’s Community Health Services Act provides for the remuneration of CHVs and provision of medical insurance for the same CHVs but does not explicitly state how community representatives in the CHC would be remunerated. The rural study county developed a Community Health Services bill in November 2020. In addition to explicitly defining the roles and responsibilities of CHCs, the bill proposes that the County should allocate 10% of the annual health budget to the delivery of community health services. Both legal frameworks in the urban and rural study counties also stipulate how community health services, including the activities of CHCs, shall be financed by county governments. The Community Health Services Bill for the rural study county explicitly stipulates that CHC members should not receive any remuneration or honoraria for the performance of their duties. The national Community Health Services Bill (dated April 2020), which was still being debated in the national Parliament (Senate) at the time of this study, makes a provision for counties to enact context-specific legislation on the remuneration of CHC members. Interview participants in the urban county reported that the legal frameworks were developed to facilitate the remuneration of CHVs to motivate them and reduce attrition. At the same time, primary health workers who participated in our interviews reported high attrition of CHC members, and part of them becoming CHVs, because they did not receive any financial incentives as CHC members. This contributed to the dormancy of CHCs in both rural and urban settings.

“[laughs] *when the formation of the units came, the CHCs were not factored in, actually the stipend was not… aahh… they were not factored in, the CHCs role is to mobilize these resources that the CHV would use, so they are seen as managers. I don’t know whether managers are not supposed to be motivated,* [laughs] *I don’t know, yeah.”* **(Female Primary Health Worker, rural county)**

Another primary health worker said:

*“… though I have not read the bill, I would have preferred they sort out the issue of CHC so that… we can retain them, you know there is no… at times they become dormant because they feel that the CHVs have been given more priorities as in, they are getting stipend and you find any other activity that comes, just values CHVs.”* **(Female Primary Health Worker, urban county)**

Insomuch as the legal framework for CHCs at the national level allows for contextualization of how CHC members would be incentivized, we observed that community health policies at county level did not provide specific guidance on how CHC members will be incentivized, compared to other cadres involved in delivering community health services.

## Discussion

We conducted an analysis of the key factors that influence the implementation of CHC policies. In this section, we discuss how the focus on service delivery, role conflict within CHCs, and lack of recognition of governance roles negatively influence the implementation of CHC policies in Kenya’s sub-national health systems.

The community health strategy in Kenya has been shown to be a cost-effective approach for delivering quality maternal and child health services at the household level (Kumar *et al*., 2021). CHVs are at the center of the community health strategy because they support the uptake of primary health services and, generally, they have built trust and credibility in their communities (WHO and UNICEF, 2021). Policymakers in Kenya also view CHVs as a critical workforce that will support the achievement of the country’s ambitious universal health coverage targets. Additionally, CHVs played an essential role in the prevention and response to the COVID-19 pandemic in urban informal settlements and rural areas (Ministry of Health, 2020b; Ministry of Health, 2020c). As a result of the prominent and visible role played by CHVs in service delivery, health system actors in government and NGOs tend to focus on advancing the performance and retention of CHVs by providing them incentives, training, and supervision during service delivery (Lehmann and Sanders, 2017; Colvin *et al*., 2021; Sarriot *et al*., 2021).

This study reveals that there is no such focus on CHCs. Consequently, CHC members perceive that their services are not valued. This is consistent with findings from another study that demonstrated how policymakers, health managers, and primary health workers perceive CHVs as more important actors in community health compared to CHCs, because CHVs provide health services that are measurable and visible (Karuga *et al*., 2019a). This skewed attention on service delivery portends the risk of community members losing interest in participating in the governance of community health services. This loss of interest creates a “vicious cycle” where no additional investments are made to develop CHCs as mechanisms for community participation due to lack of interest, which further downplays communities’ role in governance.

Role conflict within CHCs occurs when CHVs that are members of CHCs encounter conflicting obligations and roles. On the one hand, CHVs who are members of CHCs are expected to actively deliver health services, while at the same time, they are expected to objectively supervise fellow CHVs and participate in making decisions about the delivery of community health services. CHVs that are CHC members are also under the supervision of fellow CHC members. Beyond creating role conflict, we argue that CHVs serving in CHCs have to deal with the competing demands of both roles. This weakens the oversight role of CHCs by introducing mistrust, jealousy, and conflict between CHC members because the CHV role attracts financial and other incentives, such as medical insurance cover. Literature on role conflict in community participation in the oversight of community health services across the world is still scarce. More research is required to examine the effects of role conflict on the functionality of community-level health governance structures.

We note a contradiction in our study where health professionals and policymakers expect CHC members to serve as volunteers, but at the same time, provide incentives to CHVs whom they are required to oversee. Haricharan *et al*. (2021) argue that the lack of financial incentives and recognition of CHCs by other health system actors in South Africa contributes to frustration and high levels of attrition among community-level health committee members (Haricharan *et al*., 2021). Participation of community members in CHCs comes with opportunity costs, such as diminished income whenever they are involved in CHC activities. Lack of compensation to account for these opportunity costs may limit participation in CHCs to either elite in the community or salaried ex-officio members. When CHC members are not incentivized, they get disillusioned and drop out. High attrition rates of community representatives exacerbate the exclusion of community groups from making decisions about their health. Establishing incentive schemes for CHCs will require critical assessments on how to sustain such incentives for CHCs over time (Zakus and Lysack, 1998; Sakeah *et al*., 2021). Recent research on the factors that influence the functionality of CHCs in Kenya revealed that CHC members find it difficult to supervise CHVs because CHVs receive more training and incentives. This creates an odd hierarchy between CHCs and CHVs, which further hampers the functionality of CHC *(Submitted for publication*).

One of our study counties enacted a legal framework that is meant to legitimize CHCs. While it is expected that legal frameworks will ensure support and facilitate community participation through CHCs, our findings indicate that these frameworks provide a minimal basis for such support (Haricharan *et al*., 2021). County health authorities should go beyond the enactment of legal frameworks and outline clear budgets to support CHC operations, extensive training, and accountability processes for measuring the performance of CHCs. Without these investments in supporting the implementation of CHC policies, the full benefits of community participation may never be realized (Meier *et al*., 2012). Subsequent reviews of Kenya’s community health strategy will need to address the membership of CHCs to address the risks of role conflict among CHC members who are expected to provide services and oversee community health services at the same time.

We anticipate that our findings will inform counties in Kenya and other similar settings on how they can steer the implementation of policies that promote community participation in overseeing community health services through CHCs. For the successful implementation of CHC policies, county health managers need to clearly define the roles and responsibilities of these community-level governance structures to all community health actors so that they are not overlooked during the implementation of community health programs. Beyond legitimizing CHCs through legal frameworks, county health managers should allocate adequate financial and operational support for the dissemination of CHC policy content, training, and supportive supervision to primary health workers (Karuga *et al*., 2019b; Ndima *et al*., 2015).

This study provides insights into factors that influence the implementation of CHC policies. There are, however, inherent weaknesses in our study. We conducted this study during the third wave of the COVID-19 pandemic in Kenya. All interviews were therefore conducted over the phone, which meant that the interviewer was not able to read nonverbal cues during interviews. At the same time, most health workers were heavily involved in COVID-19 and other priority public health emergencies, and this limited the number of interviews we conducted. Despite these limitations, we conducted a rigorous analysis of narratives to get an in-depth exploration of our cases.

## Conclusion

This study demonstrates a disconnect between the policy plans and the actual implementation of community health governance policies in Kenya. Our study demonstrates how community health policies in Kenya inadvertently created role conflict and competition for resources and recognition between community health volunteers involved in service delivery and those involved in the governance of community health services. It will be important to address this role conflict in the next appraisal and review of the community health strategy. County government needs to clearly define the roles and support systems for CHCs in community health policies. County governments and development partners need to intensify the implementation of CHC-related by conducting regular reviews of their performance.

## Data Availability

The dataset supporting the conclusions of this article is included within the article as an additional file.

## Supplementary material

For supplementary material accompanying this paper visit http://doi.org/10.1017/S1463423623000208. click here to view supplementary material
