# Supplementary material for: Community participation in the health system: analyzing the implementation of community health committee policies in Kenya
Source: Prim Health Care Res Dev. 2023 Apr 28;24:e33. doi: 10.1017/S1463423623000208 (PMC10156468; doi:10.1017/S1463423623000208)
Supplement: Supplementary file 1 [file S1463423623000208sup001.doc]

**Supplementary File 1**

**Details of the READ Process that was applied during the document analysis(1)**

**Step 1: Ready materials**

**Topic:** Community Health Committee Policies at National and County levels in Kenya

**Source of documents**: Ministry of Health Website, County Health Assembly Websites, County Health Department websites, Key informants. Our search yielded 21 documents. We extracted data from 12 documents that were more relevant to the study.

**Dates of Inclusion:** Included policy documents from 2006 to 2021.

Table 1: Documents included in the analysis

| **Document category** | **Year of publication** | **Document title** | **Level of policy** |
| --- | --- | --- | --- |
| **Operational documents.**  These were policies, Health Sector Strategies, official declarations | 2020 | Ministry of Health. Kenya Community Health Strategy 2020-2025. | National |
|  | 2020 | Ministry of Health. Kenya Community Health Policy 2020 - 2030. | National |
|  | 2019 | Ministry of Health. Kenya Primary Health Care Strategic Framework 2019-2024. | National |
|  | 2019 | Ministry of Health. Planning, Budgeting and Performance Review Process Guide for Health Sector: Simple Guide to MTEF for Health Sector. Nairobi, Kenya. | National |
|  | 2014 | Ministry of Health. Strategy for Community Health (2014-2019): Transforming Health: Accelerating Attainment of Health Goals. | National |
| **Legal documents** included laws, Bills, and any cooperative agreements | 2020 | Kenya Senate. The Community Health Services Bill, 2020. | National |
|  | 2019 | [Urban] County Community Health Services Act 2019. | County |
|  | 2019 | Hansard Report Second [Urban] County Assembly – Third Session 18 Sept 2019. | County |
|  | 2019 | Hansard Report Second [Urban] County Assembly – Second Session 11 Sept 2019. 2019. | County |
|  | 2020 | [Rural] County Community Health Services Bill, 2020. | County |
|  | 2012 | Ministry of Health. Handbook for Community Health Committees. | National |
|  | 2006 | Ministry of Health. Community Health Strategy | National |
|  | 2007 | Community Strategy Implementation Guidelines  For Managers of the Kenya Essential Package for Health at the Community Level | National |

**Step 2: Extraction of data**

Table 2: Consolidated data extraction

| **Meaning unit** | **Condensed meaning** | **Theme** |
| --- | --- | --- |
| 1. **Ministry of Health. Kenya Community Health Strategy 2020-2025.** | | |
| Leadership and governance of community health units is under the direct mandate of CHC. These committees have executed their leadership and oversight functions as defined in the Strategy for Community Health 2014 – 2019 (Page 10 -11) | Disconnect between policy and reality about CHCs playing their role in providing leadership and oversight functions | Evolution of policy content |
| However, during this execution there was low coverage of CHCs, low motivation and poor contextualization of the community health strategies which limited the execution of the functions by the CHC.  **1. Low motivation by CHC**  a. The committee members felt overlooked during the implementation of community services especially in campaigns or other activities.  **Poor contextualization of the previous health strategy**  a. Sub optimal dissemination and utilization of the Community Health Strategy by only about 58% of the counties.  b. The implementation process of the strategy is fragmented and lacked strong inter-sectoral collaborations for joint advocacy and/or resource mobilization. | Policy document identifies that challenges faced by CHCs | Perceived value of CHC among health system actors |
| Inadequate resource allocation to community health has long been a limitation to implementation of previous strategies. Although 62% of counties reported that resources were allocated for community health, only 48% reported that the resources had been disbursed from the county treasury.  The resources provided were for activities ranging from support supervision, CHV feedback meetings and provision of supplies and commodities. It was further noted that there was heavy reliance on partners for implementation of community health services due to inadequate resources and unclear financing mechanism. (Page 11-12) | Actors involved in budgeting for health did not always disburse funds for community health in approximately half of the counties | Perceived value of CHC among health system actors |
| Challenges experienced over financing for community health include:  **1. Funding limitations**  a. Programmatic cost for community health at national and county level has been funded by Partners.  b. Partner support is heavily skewed to geographical regions and certain vertical programs. | Non-state actors play a prominent role in financing community health.  Few counties have developed legal frameworks for implementing community health | The perceived value of CHC among health system actors |
| **2. Legislation to support financing community health services.**  a. The absence of a legal framework is a barrier to county allocation of resources for community health services. The Community Health Services Bill 2020 that is currently before Parliament is envisaged to provide this legal framework.  b. Some counties, e.g. Turkana, have already passed their Community Health Services Bill, 16 counties have Bills in draft forms (Page 11-12) | Lack of legal frameworks impedes implementation of CHC policies | Process of developing legal frameworks |
| **Strategic Direction 1:** Strengthen management and coordination of community health governance structures at all levels of government and across partners  This strategic direction focuses on the strengthening of the CHCs, advocacy at all levels for community health and provision for legal frameworks for the funding and sustainability of community health services.  1.1. Strategic Objective 1: Review and institutionalize functional community health committees  Key interventions  1.1.1. Review, redesign and disseminate community health committee (CHC) guidelines in line with composition and selection criteria  1.1.2. Review training manual for CHCs  1.1.3. Involve sub-county health management to reconstitute community health committees for ownership of all community-led health activities | Clear policy expectation from the national MoH on how to strengthen CHCs | Evolution of policy content |
| **Strategic Objective 3: Strengthen performance monitoring mechanism for community health governance structures**  **Key interventions**  1.3.1. Develop and operationalize an M&E structure for CHC performance  1.3.2. Develop an accountability framework to assess the CHC functionality  1.3.3. Conduct bi-annual meetings to review resource allocation and utilization  1.3.4. Conduct quarterly review meetings on CHC performance | National level MoH has clear policy objectives on how to monitor the implementation of CHC policies in the country | Evolution of policy content |
| **Strategic Objective 4: Strengthen legal frameworks and legislations to support the delivery of community health services**  **Key interventions**  1.4.1. Advocate for finalization and enactment of the national community health services bill into law  1.4.2. Develop a regulation framework for the implementation for Community Health Services Act  1.4.3. Advocate for counties to develop and pass community health services bills (Page 15) | National level MoH provides a clear plan on how to advocate for the development of legal frameworks for CHCs in the counties | Process of developing legal frameworks |
| Implementation framework (FY 2020/2021 to 2021/2022)   - Review, redesign and disseminate CHC guidelines in line with the new composition and selection criteria - Review training manual for CHC - Involve county health management to reconstitute community health committees for ownership of all community-led health activities - Develop and operationalize an M&E framework for CHC performance - Develop an accountability framework to assess the CHC functionality - Develop a regulation framework for the implementation for Community Health Services Acts   FY 2022 – 2023   - Advocate for counties to develop and pass community health services bills   Across 5 years   - Conduct quarterly review meetings on CHC performance | MoH provides a clear timeline for strengthening CHCs | Evolution of policy content |
| **Unstainable financing plan for SG 1**  **SD 1:** Strengthen management and coordination of community health governance  structures at all levels of government and across partners (19M +11.3+9.3+12.2+10=61.8) (page 37) | MoH committed a budget for strengthening the management and coordination of CHCs across the country | Evolution of policy content |
| **Monitoring & Evaluation Framework**  **Strategic Direction 1: Strengthen management and coordination of community health governance structures at all levels of government and across partners**   - Number of counties where the reviewed and redesigned CHC guidelines have been disseminated and where CHCs have been trained on the use of the revised guidelines (10+20+30+40+47) - Number of counties where the M&E structure for CHC performance has been adopted and operationalized Number of counties in where CHCs hold quarterly community review and planning meetings - Number of counties in which CHCs are functional | MoH developed a framework for monitoring progress in the strengthening of CHCs | Evolution of policy content |
| Stakeholder roles and responsibilities  The CHC shall oversee the coordination and management of the community health units within their jurisdictions.  In doing this, the CHC shall:   - Provide leadership to the CHU in the implementation of community health services - Prepare and present the CHU annual work-plans and operational plans to the link facility health committee - Plan, coordinate and conduct community dialogue and health action days - Hold quarterly consultative meetings with the link facility (page 47) | Clearly defined roles for CHCs. Roles are not as ambitious as in the earlier documents | Evolution of policy content |
| 1. **Ministry of Health. Handbook for Community Health Committees. 2012.** | | |
| Community Health Committees (CHCs) are key for **providing an appropriate and supportive social environment** for the work of Community Health Workers (CHWs) and Community Health Extension Workers (CHEWs). | MoH recognizes CHCs as important structures for delivery of community health services | Evolution of policy content |
| Community Health Committees do this by taking responsibility for leadership, governance oversight and coordination at the community level. They also have the responsibility of mobilising communities for involvement in health-promotive and disease-prevention activities. **To enable Community Health Committees (CHCs) to be effective and efficient, the need for appropriate training was clear.** | MoH recognizes the need for training CHCs | Evolution of policy content |
| Roles and responsibilities of CHCs.  1. Provide leadership and governance oversight in the implementation of health and related matters in community health services at level 1  2. Prepare and present to the Link Health Facility Committee (and to others as may be needed) the community Annual Operational Plan (AOP) on health related issues at level 1  3. Network with other sectors and developmental stakeholders towards improving the health status of people in the Community Unit, e.g. Ministries of Water, Agriculture, Education, etc.  4. Facilitate resource mobilisation for implementing the community work plan and ensure accountability and transparency  5. Carry out basic human resources and financial management in the community  6. Plan, coordinate and mobilise the community to participate, along with themselves, in community dialogue and health action days through social mobilisation skills  7. Work closely with the Link Health Facility Committee to improve the access of the CU to health services  8. Facilitate negotiations and conflict resolution among stakeholders at level  9. Lead in advocacy, communication and social mobilisation  10. Monitor and evaluate the community work plan including the work of the CHWs through monthly review meetings  11. Prepare quarterly reports on events in the CU  12. Hold quarterly consultative meetings with the Link Health Facility Committee | The handbook provides for the roles of CHCs. We assume counties will customize the roles and responsibilities to suit their contexts | Evolution of policy content |
| CHC competencies:  1. Effective leadership and management skills  2. Communication skills  3. Mobilisation and management of resources  4. Networking  5. Report writing  6. Record/bookkeeping  7. Basic analysis and utilization of data  8. Basic planning, monitoring and evaluation skill  9. Performance appraisal skills  10. Conflict resolution skills | The training manual spelt out the competencies of CHCs. These competencies seem very advanced for CHC members in the areas where community health services are provided i.e. rural settings and urban informal settlements. These competencies require to be aligned to the community health strategy. | Evolution of policy content |
| However, in each CU, there are more than ten CHWs as each CHW works in only a part of the entire Community Unit (CU). Without any group taking on overall leadership and governance roles in the Community Unit**, there was lack of coherence in the entire CU as to what exactly was happening in the community healthcare services. The vacuum from lack of those with leadership and governance role for the whole CU became clear to implementers**, both within government and non-governmental organisations.  These implementers then began to establish Community Health Committees (CHC) in a piece-meal manner to play this overall leadership role within the Community Unit. | CHC structures were an afterthought, 5 years after launch of the community health strategy | Lack of clarity on how to implement policy on CHC |
| While this was a move in the right direction, each group was “doing its thing” more or less according to its whims! Some implementers trained their CHCs for a day. Some trained them for 2 days and so on. Furthermore, the content that the CHCs were trained on greatly varied. By the end of 2010, the need for clarity on the roles of CHCs was recognized. | Implementation of CHC training | Lack of clarity on how to implement policy on CHC |
| Also recognised was the need for guidance on content and on the  modality for training activities for CHCs. The leadership of the Ministry of Public Health & Sanitation approached development partners for financial and technical support for the development of training materials to ensure that the CHCs would have responsibility for the leadership and governance roles at the community level. The positive response from Management Sciences for Health (MSH) supported by USAID was indeed welcome. | USAID and MSH played a key role in supporting the development of the CHC training manual | The perceived value of CHC among health system actors |
| Process of establishing CHCs  1. The CHEW is the Technical Adviser and Secretary to the CHC and shall oversee the operations of CHCs and CHWs in the CHEW's area of responsibility. The CHEW facilitates referrals from the CU to the referral health facility  2. The CHC shall be the first body constituted in the operationalisation of the CHS in a Community Unit and shall come before the selection of CHWs  3. The CHC chooses its own Chairman  4. CHCs **should have at least one CHW (and a maximum of 2 CHWs) in its membership who will be selected by the other CHW**s in the Community Unit  5. **One CHW member of the CHC shall hold the position of treasurer**  6. If a **CHC member should be selected to be a CHW, he/she ceases to be a member** of the CHC unless he/she is one of the two selected by the other CHWs  7. The **chairman of every CHC should be a member of the Link Health Facility Committee**  8. The CHC and CHWs shall work together on implementing Community Dialogue Days and Community Action Days (quarterly). Further, the CHC may call all CHWs to a meeting in case of need such as the occurrence of an epidemic in the area. | The training manual spells out the process of establishing CHCs and the composition of CHCs. The manual seeks to standardize the membership of CHCs | Evolution of policy content |
| The Committee shall be made up of 11 - 13 members from the Community Unit of whom **at least one third shall be women with one of them being from a woman's group**. There shall also be one each from **faith community, youth and from people with disabilities**. The rest shall be selected to ensure equality of representation of areas/ villages in the community but not exceeding 11 members in the CHC. After forming **the CHC, the CU may co-opt 3 – 5 members** on the basis of specific qualities which the CU considers essential but which are not in the CHC. Community Health Committees will be selected on the following bases, ensuring an odd number of members in each CHC:  1. Adult of sound mind and good standing in the community  2. He/she should be a resident in the area  3. Ability to read and write at least in one language: local or national  4. Elected/selected from the sub-location baraza  5. Demonstrated role model in positive health practices  6. Demonstrate leadership qualities  7. Representative of an interest group in the community, e.g. village, women who should be at least 1/3 of the CHC, faith communities, youth, disabled. The CHC shall ensure equality of representatives among the villages without going beyond 11 members  8. Demonstrated commitment to community service  9. The term of membership in a CHC is 3 years renewable once for a maximum of two terms unless the community specifically decides otherwise  It must be ensured that at any one time at least one third of the CHC members are continuing members unless the CU decides otherwise.  From the foregoing information, it is clear that the CHC team members have a very important role to play and this Handbook is for the easy reference of each member so that each is equipped to play the important role expected of them. | Ministry of Health had standardized expectations on CHC members across the country | Evolution of policy content |
| Module 1: Leadership  The purpose of this module includes establishing an environment in which CHC members are comfortable enough with one another to work together as a team, for the CHC members to understand  their roles and responsibilities and to get equipped for the practice of leadership in the Community Unit.  Unit 1: Roles of CHC and CHV, formation, eligibility criteria.  Unit 2: Factors that promote /hinder health and development  Unit 3: practice of leadership & management:  *“Under leadership, there are the functions of scanning, focusing, aligning and mobilising. Under management, there are the functions of planning, organizing, implementing and monitoring*  *and evaluation.”*  *“What motivates employees to go to work each morning? Social psychologist Douglas McGregor of Massachusetts Institute of Technology expounded two contrasting theories on human motivation and management in the 1960s: The X Theory and the Y Theory*”   - Comparison of X and Y leadership - Application of X and Y styles   Leadership styles  Unit 4: How use the challenge model  8 steps in using challenge model  Development of vision and mission | Training approach is lecture based lasting 1-2 hours  Language is too technical  No practical sessions | Training content for CHCs is too difficult to comprehend |
| Module 2: Governance in the context of community health  Unit 1: Definition and principles of governance, governance roles of CHC members  Unit 2: Definition of PHC  Unit 3: Community Health Strategy and Kenya’s health policy / strategy | Practical skills not imparted. There are no worked examples  Complex content from management theory  Too much content for 7 days  Pedagogy guidance – appears to be lecture based -1 hour /2 hour lectures back to back  Does not consider that CHC are adult learners. In some cases members may have limited education  Lacks room for critical reflection and thinking | Training content for CHCs is too difficult to comprehend |
| Module 3: Role of CHC in effective communication, advocacy, networking and social mobilization  Unit 1: Roles of CHC in effective communication  Unit 2: Roles of CHC in advocacy  Unit 3: Roles of CHC in networking and partnership development  Unit 4: role of CHC in social mobilization –steps, strategies to social mobilization, techniques to social participation, principle and approaches to social mobilization | 1 hour lectures. No worked examples  No Practical sessions | Training content too difficult to comprehend |
| Module 4: personnel management  Unit 1: Definition of HRM,  *“A strategic and comprehensive approach to managing people, the work-culture and environment. Effective human resource management enables people to contribute effectively and productively to the overall goal of the group and in the accomplishments of the group's objectives and goals.”*  *Levels of management, Introduction to personnel management , elements of HRM”*  Unit 2: Performance appraisal (definition of performance appraisal, importance of PA,  Definition of performance appraisal *“A performance appraisal is a part of guiding and managing career development. It is the process of obtaining, analysing, and recording information about the relative worth of a person's skills.”*  Unit 3: Conflict management and resolution in the CU  Definition of conflict resolution, steps in conflict resolution, negotiate /address conflict | 2 hour lectures 8:30 – 4:00 pm  No tools for performance appraisal, no practical, no explanation on how to perform appraisal)  Lacks vignettes to facilitate understanding  Complex language e.g., pre-caucus mediation, arbitration | Training content for CHCs is too difficult to comprehend |
| Module 5: resource mobilization & financial management  **UNIT 1:** The Role of CHCs in Resource Mobilization for the Community Unit  Steps in RM, factors to consider, strategies for RM  **UNIT 2:** Proposal Writing  Structure of a proposal  **UNIT 3:** The Role of CHCs in Financial Management in the Community Unit | 2 hour lectures (8:30 am  No practicals  Not applicable in real life  Mentorship  Proposal format is academic  Content on financial management is copied from text books and not tailored for CHC  No practical skills | Training content for CHCs is too difficult to comprehend |
| Module 6: community health information system | Lectures range between 1 -2 hours  No Practical sessions | Training content for CHCs is too difficult to comprehend |
| Module 7: Monitoring and evaluating | Technical content too much for a 2 hour session | Training content for CHCs is too difficult to comprehend |
| 1. **Ministry of Health. Community Health Policy 2020 - 2030.** | | |
| The objective of this section is to secure effective leadership and governance for community health services. This objective also guides the formation, maintenance and governance of various community health structures critical for effective management and governance for an effective community health services platform. Community health services delivery shall be guided by a well-functioning community health governance system described below. (Page 10) | The Ministry of Health provides guidance on the formation of CHCs | Evolving policy content |
| **Formation and setting up of a community Health unit**  The formation of the CHU shall follow a structured community entry process which begins with awareness creation, situation analysis, and formation of linkage structures, training teams and establishing the monitoring and evaluation mechanism. The entire process shall be overseen by the CHA representing the county health management team and partners, in collaboration with the county administrative structures. The process shall entail:   1. Creation of awareness among county stakeholders, including the county/subcounty health management teams, health facility managers and local leaders and social mobilization of the community members to attend barazas for the selection of their CHC and CHVs. 2. In a baraza setting, CHCs are selected then trained on their roles and responsibilities using the nationally approved CHC training curriculum. 3. Upon completion of training, CHC members organise a baraza for the selection of CHVs. CHVs are then trained on their roles and responsibilities using the nationally approved CHV training curriculum. (Page 10) | Ministry of Health provides a clear vision for the establishment of CHCs | Evolving policy content |
| **Governance of Community Health Services -Community Health Committee (CHC)**  The coordination and management of the CHU and its workforce shall be done by a CHC, a group of members selected by the community. The committee shall include:   - A prescribed number of which not more than two thirds shall be from same - gender - Representation from religious and Cultural groups within the context - Representation from youth and people with disabilities - Members must reside in the community they are selected to serve. They will serve a three-year term that is renewable once, unless agreed by the community. - The CHC shall choose its chairperson, and shall have at least one, and at most two CHVs. If a member of the CHC is selected to be a CHV, they cease to be in the CHC unless representing CHVs. The CHA shall be the technical advisor and secretary to the CHC. - The treasurer shall be a CHV. - The chairperson shall become a co-opted member of the link health facility committee. - The CHC shall be the first organ to be constituted in the establishment of a CHU. | The Ministry of Health provides guidance on the composition of CHCs | Evolving policy content |
| The roles and responsibilities of the CHC shall include:  • Provision of leadership and oversight in the implementation of health and other related community services  Preparation and presentation of the CHU annual work-plans and operational plans to the link facility health committee  • Planning, coordinating and conducting community dialogue and health action  days  • Working with the link facility to promote facility accountability to the community  • Holding quarterly consultative meetings with the link facility  • Creating an enabling environment for implementation of community health  services  • Resource mobilization for sustainability (Page 12 ) | The policy stipulates the roles and responsibilities of CHCs | Evolving policy content |
| 1. **Ministry of Health. Strategy for Community Health (2014-2019): Transforming Health: Accelerating Attainment of Health Goals. 2014.** | | |
| These committees have played a key role in increasing awareness of health rights, raising social accountability, and growing advocacy efforts. The CHC roles and structures are also clearly defined. (Page 6) | This strategy identifies the roles played by CHCs. | Evolving content of CHC policy |
| However, coordination mechanisms and the linkages between CHC and Health Facility Management Committees (HFMC) are weak. Irregular stakeholder forums coupled with lack of guidelines for CHC members on their tenure, poorly oriented staff, absence of remuneration for work done, poor feedback and information flow and use, the split of the Health Ministry into two parts in 2008 (now reintegrated), CHCs’ demands for stipends, and frequent transfers and/or removal of focal persons and CHEWs greatly affects leadership and governance at the community level. (Page 6) | The strategy was informed by insufficient evidence on CHCs | Evolving content of CHC policy |
| The existence of the CHS focal persons at county and sub-county levels, the approved scheme of service, county health stakeholder forums for advocacy and sensitization, and the recognition of the CHCs present opportunities for strengthening community leadership and governance (Page 6) | Strategy was informed by insufficient evidence on CHCs | Evolving content of CHC policy |
| Community health committees (CHC)   1. Understand and own the strategy 2. Actively contribute to the implementation of the strategy 3. Evaluate the strategy implementation and provide feedback to stakeholders at the community level, including the link health facilities Implement relevant aspects of the strategy, e.g., provide the work force Provide social accountability to community members by attending dialogue days and sharing community issues and participating in action days 4. Participate in annual work plan development at community level 5. Participate in health data collection and utilization 6. Advocate to the county leadership for various community health needs 7. Generate information towards the future review process (Page 19) | The strategy spelt out responsibilities of the CHCs. These roles are clearer that those in the earlier versions | Evolving content of CHC policy |
| 4.2.3 CHC Gazetted  4.2.3.1 Advocate for a legal framework for gazettement of Community Health Committees (CHCs)  4.2.3.2. Advocate for adoption of community units as spending units (Page 31) | Development of a legal framework for CHCs has been in the strategy since 2014 | Development of legal frameworks |
| 1. **[Rural] County Health Services Bill, 2020** | | |
| **Object of the Act.**  **3.** The objects of this Act are to —   1. provide the framework for the performance of the powers and functions of the County Government in respect of promotion of primary health care at the community level; 2. provide a **framework for the effective coordination, governance, regulation and delivery of integrated, comprehensive and quality primary health services at the community** and household level; 3. provide for the **establishment of community health units** within the county health system for the effective, efficient and sustainable delivery of community health services; 4. enhance household and community access to basic health services; 5. provide a framework for the coordinated implementation of national and county policies and standards for the realisation of the right to health at the community level; 6. entrench the role of community health workers and/or volunteers as frontline workforce in the provision of basic health care at the community and household levels through a range of health, social action and community empowerment activities; 7. provide a framework for planning and sustainable financing of community health services; 8. provide a mechanism for the monitoring and tracking of national and county health indicators and progressive realization of the right to health at the community level; and 9. provide institutional framework for strengthening the community health system and community participation in the implementation of community health actions, programmes and services. | Bill proposes to create a legal framework for coordination of community health services | Contents in the county level legal framework for CHCs |
| **10.** (1) There shall be appointed or designated a director within the county public service to be in charge of community health services.  (2) The Director shall be the head of the county community health function and will be responsible to the Chief Officer for the overall coordination of all community health services and activities within the county.  (3) The functions of the director of community health services in connection with the administration of this Act shall be to -   1. oversee the implementation of quarterly programmes for—   (i) community health dialogue days; and  (ii) health action days with respect to community health units;   1. facilitate and enable community health units to access to information necessary for their effective operations and decision making; | Bill proposes the establishment of a Director for community health to oversee the implementation of quarterly programs that will be run by CHCs | Contents in the county level legal framework for CHCs |
| **Establishment of community health units.**  **14.** (1) The County Executive Committee member shall establish, in so far as practicable and efficient to do so, such number of community health units as the first level of the county health system in accordance with the procedure outlined in the First Schedule of this Act.  (2) The community health unit shall cover a defined geographical area with defined number of households and population as the county executive committee member shall determine.  (3) The community health units shall be governed by the **community health committees, administered by a community health assistant**, and served by such number of community health workers or volunteers as the county executive committee member, in consultation with county public service board, shall determine.  (4) The community health units shall so far as practicable, promote access to basic health services and ensure effective, efficient, equitable and sustainable delivery of integrated, comprehensive and quality community health services at the household and community level by:-   1. ensuring access to, and provision of basic health care services at the household and community level; 2. facilitating and coordinating the provision of community based health services and programmes including community mobilization, empowerment and social; 3. fostering community empowerment and development initiatives and promoting community ownership of community based health services and programmes; 4. facilitating and conducting quarterly community dialogue and feedback on community health and outreach activities according to guidelines; 5. facilitating regular community and household health assessments or diagnosis and surveillance of disease outbreaks, environmental hazards, and other health threats at the community level; 6. leading community based health campaigns including promoting good sanitation and hygiene behaviors, adoption of improved sanitation technologies and household water safety; 7. preparing, maintaining and regularly updating community health profiles, household health registers and inventory of community-based health services delivered in the community; 8. ensuring effective functional linkages and referrals between the community health units and link facilities according to service guidelines and standards; 9. facilitating inter-sectoral/multi-sectoral coordination at the community level to address social determinants of health 10. generating local solutions to local health problems; 11. creating public awareness and advocacy about the essentials of better personal and community health including environmental health, sanitation and hygiene; 12. facilitating development of community health unit work plans and budgets in accordance with prescribed guidelines; 13. sensitization of communities on their health and related rights and responsibilities; 14. promoting public participation in county planning, budgeting and other decision making processes at the community level; 15. promoting local resource mobilization initiatives for community health promotion and delivery of community health services; 16. distribution of IEC materials, standard formats and tools for data collection and reporting and others resource materials according to guidelines; 17. community based procurement and distribution of community health commodities and supplies according to guidelines 18. monitoring all community health activities conducted at the household and community level; 19. preparing and submitting monthly, quarterly and annual community health unit reports in accordance with prescribed guidelines; | Formation of community units guided by a legal framework. Proposed bill stipulates that CHCs will govern CUs where services will be provided, empowerment, dialogue, health assessments, etc. | Contents in the county level legal framework for CHCs |
| **Establishment of community health committee.**  **16.** (1) The Executive Committee Member shall by notice in the Kenya Gazette, establish, for each community health unit, a community health committee which shall be a voluntary body.  (2) The membership and procedures of a community health committee shall be in accordance with the Second Schedule of this Act.  (3) The County Executive Member shall promulgate regulations in respect of the establishment, duties, functions, and operations of the community health committees, and in particular setting out —   1. the procedure for the nomination and election of members of the committee; 2. the term of appointment of the members of a committee; 3. such other criteria for nomination or election as the county executive committee member shall consider appropriate; 4. terms and conditions for appointment of the members of the committee; 5. grounds and procedure for the removal and replacement of a member of the committee; 6. the procedure for the conduct of the business and affairs of the committee and for the convening of public forums by the committee; 7. procedure for dissolution of a community health committee; and 8. such other matters as the county executive committee member may consider necessary for the effective functioning of the community health committees. | Formation procedures, terms of appointment, term limits, removal, dissolution and conduct of business of CHCs is now guided by the proposed bill | Contents in the county level legal framework for CHCs |
| **Functions of the community health committee.**  **17.** (1) The Community Health Committee through mutual assistance and cooperation shall:   1. provide local leadership, oversight and representation of community interests in the affairs of the community health unit; 2. provide such advice and assistance that the community health assistant and team of community health workers or volunteers may need; 3. coordinate and work with the local administration to mobilize the community, households and residents to participate in planning, budgeting and decision making; and 4. strengthen inter-sector collaboration and linkages between the community and the county health system 5. support and facilitate regular dialogue between the community and the health service providers, public forums, community dialogue and action days; 6. support outreach and referral activities of the community health unit; 7. participate in planning, implementation, monitoring and evaluation activities of the community health unit; 8. facilitate mobilization of community based resources to support community health unit plans and activities; 9. oversee management of community health unit funds including community health grant on behalf of the Chief Officer; 10. receive and approve community health unit plans, budgets, quarterly activity and financial reports; 11. report to, and advise the director on all matters affecting the health and wellbeing of the population and community; and 12. perform such other functions as the County Executive Committee Member may deem necessary.   (2) The functions of community health committee shall be inclusive and shall not in no manner be construed to limit any other function lawfully vested in it in respect of community health services by the county government.  (3) The community health committee shall organize annual public forums for the purpose of reviewing the activities of the community health unit and seeking community input on the next financial year’s plans and priorities.  (4) The Chief Officer shall provide the necessary administrative and financial support to enable community health committees efficiently and effectively perform their functions under this Act. | The Bill proposes that the Chief officer will provide necessary administrative and financial support for CHCs to perform their roles | Contents in the county level legal framework for CHCs |
| **Community health unit plans and budgets.**  **46.** (1) A community health unit shall develop and implement a community health plan and budget for each fiscal year.  (2) The community health unit plan and budget shall include priorities, programs and activities for improvement of community and population health and wellbeing identified and recommended by the community through community consultations and public participation forums.  (3) The county executive committee member shall ensure that community health plans and budgets are considered and factored in the preparation of the county departmental annual plan and budgets. | Proposed bill stipulates central role of community units in the development of their annual plans and budgets to be included in the county budgets and the procedure to be followed | Contents in the county level legal framework for CHCs |
| **Financing of community health services and programs.**  **47.** (1) The County Executive Committee member shall mobilise resources and allocate adequate funds necessary for the effective delivery of community health services.  (2) Notwithstanding the generality of subsection (1), the community health services and programs may be funded through-   1. appropriation by the county assembly for that purpose; 2. allocation and disbursement from the Migori County Health Services Fund established under Section 48 the Migori County Health Services Act No. 3 of 2019 for that purpose; 3. ward development fund for that purpose; 4. national government conditional or unconditional grants for that purpose; 5. donor funding; 6. grants, gifts or donations or any other contribution for the purpose; and 7. any other lawful source of funding.   (3**) For the purposes of subsection (1)(a), the County Health Department shall allocate at least 10 percent of the total approved departmental annual budget for community health services in every financial year**.  (4) Monies allocated and disbursed under subsection (2) (b) shall be utilized solely for provision of community health services contained in the proposal approved by the Fund Board.  (5) The County Executive Committee member shall establish a designated bank account for community health services to which all funds or revenue received for community health services shall be paid pursuant to section 109(2)(a)(b) of the Public Finance Management Act No. 18 of 2012.  (6) Disbursements out of the designated account shall be for specific county and community health unit programmes or projects. | Bill clearly identifies the potential funds that may be used to finance community health services  Bill proposes that 10% of annual health budget of the county be should be for community health services | Contents in the county level legal framework for CHCs |
| **Financial management by community health units.**  **49.** (1) Each community health unit established pursuant to this Act shall open and manage an account with a credible bank or financial institution.  (2) Each community health unit shall:   - 1. keep accurate financial and accounting records of all funds received and expenditures   2. make financial and accounting records available to the chief officer, community members and other stakeholders upon request;   3. have the treasurer, chairperson and secretary as signatories to the bank accounts;   4. maintain receipts, labour registers, and other supporting documents for all expenses;   5. provide quarterly reports to the community on the current financial and physical status of the projects/activities being implemented through community forums/meetings or other agreed means;   6. maintain a register of all cash, labour, and material contributions from the community, valued at local market rates; and   7. timeously submit quarterly activity and financial reports to chief officer through the director.   (2) The record of the amounts received and spent by each community health Unit shall be submitted to the director and chief officer for health within thirty days after the close of the financial year together with a copy of bank statements. | Bill proposes the financial management roles that will happen in Community Units | Contents in the county level legal framework for CHCs |
| CHU legally established via legal/gazzete notice by the County Executive Committee Member | The bill stipulates the legality of Community Units, which are governed by CHCs | Contents in the county level legal framework for CHCs |
| - CHU governance by a Community Health Committee consisting of 9-13 members democratically appointed or elected by the community members. - The membership of the CHC must not have more than two thirds of one gender represented in the Committee. - The youth, persons with disability and other marginalized groups in the community are represented in the CHC - Members of the CHC are gazzeted by the County Government. - Members of the CHC are guided by the values and principles of governance, leadership and integrity under the Article 10 and Chapter Six of Constitution of Kenya. - CHU and CHC governance and operational guidelines.   CHCs hold regular quarterly meetings | This bill provides guidance on the composition of CHCs and stipulates that they should be gazetted by the County Government | Contents in the county level legal framework for CHCs |
| - CHU secretariat headed by a community health assistant - 2 -3 administrative support staff - A basic financial management and accounting system - A basic commodities management and security system - An inventory of assets - A basic asset management system - Maintenance plan - Community based procurement system - Regular financial reporting according to guidelines   The financial records of the CHU are open to public scrutiny | Bill proposes that CU will be provided administrative support by a secretariat | Contents in the county level legal framework for CHCs |
| 1. **Ministry of Health. Planning, Budgeting and Performance Review Process Guide for Health Sector: Simple Guide to MTEF for Health Sector, 2019** | | |
| This MTEF Process Guide is developed to assist Community Health Committees, Health Facility Management Teams, Sub-County and County Health Management Teams, and National Departments by guiding them to understand and practice MTEF process. It will also help in aligning and harmonizing the planning, budgeting and reviewing processes (Page 3)  The Guide is expected to assist all the players in the health sector in order to understand the reviewing, planning and budgeting processes following the MTEF cycle in which we believe would contribute to improved service delivery and raise the health status of all Kenyans and track the progress of UHC. (Page 5) | The MoH seeks to harmonize the process of planning, budgeting and reviewing processes for CHCs | Evolving content of CHC policy |
| ***Quarterly routine 1 (Community Action)*:**  1. **CHAs** organize **the Dialogue Day** with **CHWs** and **CHCs** in the communities to discuss issues, give their views, and agree on the action to be taken.  2. **CHAs**, with **CHWs** and **CHCs** put into action what they have discussed and agreed on the Dialogue Day (including but not limited to: organizing **Community Action Day**). Page 15  Monthly budgeting activities – February  ***Starting AWP formulation:***  1. **CDOH** conducts Public Consultation to share the priorities for FY(X+1) with the Public.  2. **CHMT** calls for AWP formulation meeting with **SCHMT**, **Hospitals, Facilities and CUs**, and distributes **AWP templates**  3. **SCHMT, Hospitals, Facilities and CUs** fill their own **AWP template**.  4. **SCHMT** collects **AWPs** from **Hospitals, Facilities and CUs**. | There is guidance for how CHCs should contribute to the development of quarterly and annual CU work plan | Evolving content of CHC policy |
| 1. **[Urban] County Assembly Hansard, 18th September 2019** | | |
| **Hon. *[omitted]*:** Mr. Chairman Sir, I beg to move that the committee do report to the Assembly its consideration of the [Urban] County Community Health Services Bill 2019; Assembly Bill number 5 of 2019 and its approval thereof without amendments (Page 4) | The Bill was approved during the third reading without amendments | Development of legal framework that addresses implementation of CHC policy |
| **Hon. *[omitted]*:** Thank you Hon. Speaker. I wish to move that the [urban] County Community Health Services Bill, number 5 of 2019 be read a second time.  Hon Speaker, the objective of the Bill is to provide for the establishment and regulation of community health unit within the county for effective, efficient and sustainable delivery of community based health services and establish the necessary institutional and regulatory mechanism to ensure functionality of community units in empowering households in health and for connected purpose.  Hon. speaker, the Bill further purposes to give effect to the provision of Article 43 (1) of the Constitution. Hon. Speaker, if this Bill becomes law, the, health department will slowly shift from curative care to preventive care. This is because Hon. Speaker, once the units are established in our wards, the whole [urban county], the CHVs will be going door to door collecting data on daily occurrence especially on outbreaks of communicable diseases and disseminate the information upwards e.g. to the public health officers which in return will be going to the ground and suppress the disease before it spreads.  Hon. Speaker, it is the first time that the CHVs will get some kind of payment to recognize that they are also doing something for the community. It is the first time that the group will be wearing protective gear. The group will be taken to seminars among others.  Hon. Speaker, if the Bill gets through, the CHVs will be covered with health insurance i.e. NHIF. The relationship between the county and the health workers will be enhanced. That will also translate to service delivery to the community. Without talking much, most of the Members here are aware of how these people are suffering. The county has not been engaging these people for long. The groups are not evenly distributed. In the whole of ***[omitted]*** ward, we have just one unit. We don’t have these kind of people. So, the Bill will help the county to make sure that we have units in every ward that will be going round and collecting data as I have said. Without much ado, I wish to call my able Chair, Hon ***[omitted]***, to second. | 11 September 2019  Out of the 9 MCAs only one mentioned governance structures. Most debate was on service delivery by CHVs, their volunteerism, stipend and benefits to their stipend.  This is also an indication that most policy makers are aware of the services provision role of CHVs and are not sensitive about governance of community health services. | Development of legal framework that addresses implementation of CHC policy |
| **Hon. *[omitted]***: Thank you Hon. Speaker. I rise to second this very important Bill. As you have seen the principle object of this Bill is to ensure that we have a full establishment of community health units. We are moving from calling the Community Health Volunteers and putting them back to payroll and we will then be able to move from curative care to preventive care.  We need to improve the effectiveness and efficiency of community health workers. I believe it is now time to focus and move to preventive care from curative care. By so doing, we will be able to have a saving mechanism. We will be ensuring that we have attention to the last unit of this particular approach. I believe we have worked on this Bill. It has taken our time. The voluntary mechanisms of the health workers have taken long. These people have been giving services for free for 20 years. I want to thank the Assembly. We were able to set aside some money to give motivation to this very important component of healthcare. I want to reiterate that whatever we have given the caregivers has since been increased with about 200 million and therefore have the budgetary allocation for this particular sector. We are not yet there but I strongly believe that this will be the starting point to ensure that we will be able to motivate our people and I hope by so doing, we will be able to give back to the community. When we do this, we will be able to create employment and be able to bring health close to the community who need it most.  I want to thank the Committee of Health and we look forward that before the end of the day, we will be able to go to the next level with this Bill. For this, I propose that we move with speed and ensure that we pass this legislation. Thank you Hon. Speaker |  | Development of legal framework that addresses implementation of CHC policy |
| I want to agree more or less with the Chair that if you really allocated money which you have done as he puts it; let the Bill be passed immediately possibly by next week then it goes to the Third Reading and subsequently let us get a way that for these workers so that no one takes advantage of them. We start with those who have suffered for many years. You know in Africa at a particular moment like this, you find there are more community health workers than those who were there. We will take you to task Chair and your committee. That mother at ***[omitted]*** Eliza should be the first beneficiary of this bill. She should be proud to say that the County Assembly of [urban county] passed the budget and she was getting Ksh 5,000 per month to take her kids to school as she helps the community.  Once again thank you so much *Mheshimiwa* ***[omitted]***. We in the opposition support you fully and we support this Bill for the interests of our people. Thank you very much. |  | Development of legal framework that addresses implementation of CHC policy |
| **Hon. *[omitted]*:** Thank you. Hon. Speaker, I rise to support this beautiful Bill. I want to once again congratulate senior Hon. ***[omitted]*** being an elected leader in an area which has the highest population and comprises of informal settlements.  I happen to be working very closely with the community health workers in the entire ***[omitted]*** sub-county, we have one facility; ***[omitted]*** Health Centre which happens to be in my Ward and the great ***[omitted]*** I think it is the second biggest slum in ***[omitted]*** after ***[omitted]***. As for challenges faced by the volunteer community health workers, I can see this Bill is seriously looking and considering their interests which are the interests of the most residents of ***[omitted]***.  I am happy that at least this Bill is going to empower the structures of governance and give them an opportunity to attend workshops and trainings that will give them proper knowledge not only as volunteers but proper knowledge to deal with serious epidemics like cholera that affects us in day to day basis in the the informal settlements because of human influences. Without wasting time Mr. Speaker, this is a good bill and I support it. Thank you.  **Hon. Temporary Deputy Speaker**: Thank you very much Hon. ***[omitted]***. This House is blessed to have such brains which I admire. |  | Development of legal framework that addresses implementation of CHC policy |
| **Hon. *[omitted]***: Thank you Hon. Speaker. I want to congratulate Hon. ***[omitted]*** because there is health hazard in some of the slums and we have people who have volunteered for many years. In my Ward they have been helping in polio campaigns and attending to people who are bedridden. They don’t care about their lives but help the community and are used by the government or the county during measles outbreak or any other activity. They have been suffering a lot because they are not paid for their services. But I think with this Bill they will be given something to take home to pay for their children school fees.  I will like also to say something to our government.  Most of the governments are using these people voluntarily. They don’t appreciate what they are doing. This Bill will help fill the gaps in the health sector. Thank you, I support. |  | Development of legal framework that addresses implementation of CHC policy |
| **Hon. *[omitted]*:** Thank you, Mr. Speaker Sir. First of all, I would like to congratulate my able number one goalkeeper in CASA for bringing out this Bill. Recently, I had a meeting with these community health workers in my office. They face a lot of challenges. They have been working for the last 20 years without any pay. Eradication of things like abortion and HIV AIDS counselling have been done by these health workers. If the Assembly can pass this Bill, cases like these will decrease. I support, thank you. |  | Development of legal framework that addresses implementation of CHC policy |
| Mr. Speaker, I do not think that we will go round many blocks looking for solutions other than to make sure that such solutions which come from this House are formulated properly in the way that Hon. ***[omitted]*** has done to this Bill. We should make sure we follow it through for implementation. I will be happy to see this Community Healthcare Bill being ratified and being implemented. I am so sure it will. but when that time comes, I want to see that the community health care providers who we are called volunteer are remunerated properly. It is an avenue for job provision and it should not be taken lightly |  | Development of legal framework that addresses implementation of CHC policy |
| **Hon. *[omitted]*:** Thank you Hon. Speaker, I want to thank the Hon. Members for supporting the Bill. Mr. Speaker, there was something that was reviewed by the community of health workers during the public participation, whereby they said that when they go out to administer to the sick people in the evening and come back to their houses they find their houses closed by the landlord which is very discouraging.  They also attend to the patient but when they return home they do not have anything to provide to the children, which makes them tire to bed with an empty stomach. Therefore, I am happy together with my colleagues that soon the problem will be sorted out. I therefore, take this opportunity to thank everyone and especial the Health Committee where I sit, because they have actually worked very hard to make sure that this Bill goes through |  | Development of legal framework that addresses implementation of CHC policy |
| **Hon. Temporary Deputy Speaker:** Thank you very much *Mheshimiwa* ***[omitted]*** and I want to take this opportunity also to thank you again for the noble Bill that you have brought forth. I also represent a ward, I represent the volunteers, and I know they will be very excited to see that the Bill go through. |  | Development of legal framework that addresses implementation of CHC policy |
| 1. **Kenya Senate. The Community Health Services Bill, 2020.** | | |
| A Bill for AN ACT of Parliament to provide a framework for the delivery of community health services; to promote access to primary health care services at the community level and reduce health disparities between counties; to provide for the training and capacity building of the community health workforce.  Roles of county governments   1. monitor and evaluate the effectiveness of community health programs delivered within the respective county; 2. in every quarter, collate, analyse and disseminate   information on delivery gaps and needs  (1) Where the county executive committee  member establishes a community health committee under  section 9 (2) (b), the committee shall consist of —  (a) a community health worker nominated by  community health workers within that  community health unit;  (b) the following persons elected by the community  in a *baraza* convened by the county executive  committee member in accordance with the  respective county legislation —  (i) a woman representing women groups in that  community;  (ii) a person with disability representing persons  with disabilities in that community;  (iii) a representative of the youth in the  community;  (c) one person representing the inter-religious  organizations in the community nominated by  an inter-religious organization with the largest  membership;  (d) a ward public health officer;  (e) a community health officer who shall be an *ex*  *officio* member of the committee and the  secretary to the committee; and  (f) such other persons, not exceeding two, as may  be prescribed in the respective county  legislation. | The national-level policy seeks to provide overall guidance on the composition and representativeness of CHCs. These sections will be useful for guiding the implementation of the policy at the county level. | Development of legal framework that addresses implementation of CHC policy |
| A person is eligible for appointment as a  member of the committee if that person is —  (a) an adult of good standing with leadership  qualities in the community;  (b) a member of the community;  (c) is literate and can read and write in at least one  of the national languages or the local language;  (d) is not disqualified under any written law in  Kenya from being appointed in to a public  office; and  (e) meets such other requirements the county  executive committee member may, in county  legislation, prescribe. | The national-level policy seeks to provide overall guidance on the composition and representativeness of CHCs. These sections will be useful for guiding the implementation of the policy at the county level. | Development of legal framework that addresses implementation of CHC policy |
| In appointing the committee under subsection (3), the county executive committee member shall ensure  that not more than two thirds of the persons appointed represent one gender.  (6) Each County Government may enact county specific legislation setting out —  (a) the procedure for the nomination and election of members of the committee;  (b) the term of appointment of the members of a committee;  (c) such other criteria for nomination or election as the county executive committee member shall  consider appropriate;  (d) the remuneration and terms and conditions for appointment of the members of the committee;  (e) grounds and procedure for the removal and replacement of a member of the committee;  (f) the procedure for the conduct of the business and affairs of the committee and for the convening of public forums by the committee;  (g) such other information as the county executive committee member may consider necessary for the effective functioning of the committee. | The national level policy provides overarching guidance on how to appoint CHC memebrs at county level | Development of legal framework that addresses implementation of CHC policy |
| 1. **Ministry of Health. Taking the Kenya Essential Package for Health to the Community: A Strategy for the Delivery of Level One Services. Nairobi, Kenya: MoH; 2006.** | | |
| Role of Households and communities in governance and management of health services:  Households are expected to attend and take an active part in meetings to discuss trends in coverage, morbidity, resources and client satisfaction and giving feedback to the service system either directly or through representation | Strategy assumes that community play a passive role in community health. The strategy does not seek to empower community members with planning and management skills  No mention of community level governance structures | Evolving policy content |
| Communication strategy (advocacy) at Level 1 is left for DHMT members to advocate for support by religious, political leaders and CBOs for resource mobilization and allocation. This involves informing leaders and influential persons about aims, objectives and activities in the community health strategy  Social mobilization: DHMTs required to build capacity of CORPS and extension workers with knowledge and skills to mobilize  Interactive/Participatory communication for Level 1: Responsibility not assigned to any specific governance structure | The strategy does not seek to empower VHCs to be responsible for advocating for the community health strategy. The VHC not assigned any specific roles in social mobilization and participatory communications | Evolving policy content |
| Implementation framework – VHC not mentioned as a specific structure in the implementation framework, especially planning, implementation, feedback etc | VHCs not specifically reported in the implementation | Evolving policy content |
| Entry steps  Define clear implementation guideline (development of comprehensive message messages and materials)  Create awareness among district leaders  Form and equip district level multi-sector working groups and train the team | Formation of VHCs not clearly included in when starting off community units in the community health strategy | Evolving policy content |
| VHCs not included at the beginning of the process of establishing community health services.  Strategy does not include VHC in identification of village health priorities, development of village plans.  Village health Plans are submitted to the local health facility committee  These plans are then integrated into sub-location, location health plans for level 1. | The process for establishing community health units, identification of village health plans.  Strategy identifies NGOs, CBOs, CHEWs and CORPs and providers of technical support in the development of village plans and identification of village health priorities. | Evolving policy content |
| Linkage between Community and Health Facility & Sustainability  Health committees at divisional, locational, sub-locational and village levels can provide citizens with sufficient representation and voice in all issues affecticting service provisions  HFMC should meet monthly.  Village level: VHC will oversee operations of level one services in the village and serve as the link between the village and the household  VHC Chair report to the Dispensary committees on matters of Level One services. | The strategy placed more power to the Health facility management committee to oversee community health services.  As much as the VHC is responsible for overseeing community health services, they are required to report to the Dispensary Health Committees | Evolving policy content |
| Recruitment and Training of service providers fro level 1 service provision | Training of VHC not mentioned in the strategy. Training content for required task defined for CORPS, CHEWs and HMT members | Evolving policy content |
| Inputs and activities for implementing the Community Health | Strategy does not include inputs for VHCs in the community health strategy.  VHCs indicators not included in the M&E framework | Evolving policy content |
| 1. **Ministry of Health. Community Strategy Implementation Guidelines for Managers of the Kenya Essential Package for Health at the Community Level.** , **2007.** | | |
| The community units are organized in villages and other interest groups that are responsible for identifying and supporting the CHW. The CHWs report to the community health committee (CHC) through the community health extension worker (CHEW), who is the secretary to the committee.  All the villages within the community unit should be represented on the CHC. Since health status depends on factors beyond the health sector, coordinated action across sectors at the community level will increase efficiency in improving health outcomes. | Implementation guide recognizes the position of CHCs in the strategy. This is an amendment to the initial strategy that referred to the community level governance structure as Village health Committees | Evolving policy content |
| **Community Health Committee (CHC):** The health governance structure closest to the community is the CHC, elected in such a way that all the villages in the community unit are represented.  The CHC should be elected at the Assistant Chief’s *baraza* under the chair of the Assistant Chief. The committee is chaired by a respectable member of the community. It is recommended that a CHW should be elected treasurer and that the CHEW should be the secretary. There should be nine additional members, to include representatives of: youth, faith groups, women’s groups, NGOs, people living with HIV and AIDS (PLWHAs), people with disability (PWDs), and relevant others. At least one-third of the committee members should be women. | The guideline updated the procedure for formation and composition of CHCs | Evolving policy content |
| ***Role and Functions***  The role and functions of the CHC will include:   - Identifying community health priorities through regular dialogue. - Planning community health actions. - Participating in community health actions. - Monitoring and reporting on planned health actions. - Mobilizing resources for health action. - Coordinating CHW activities. - Organizing and implementing community health days. - Reporting to level 2 on priority diseases and other health conditions. - Leading community outreach and campaign initiatives. - Advocating for good health in the community. | The implementation guideline updated the roles and functions of CHCs. Its not clear how/why the terminology changed from VHC to CHC | Evolving policy content |
| ***Meetings and Agenda:*** The committee should meet at least monthly to receive reports from the villages to enable the CHEW to compile monthly reports for level 2 or 3 management committees. The standing agenda should include:   - Review of actions agreed on in the previous meeting and progress made in their implementation. - Review of the chalkboard records of key indicators by village (immunizations, deliveries, cases of fever and diarrhoea in children, the chronically ill, use of insecticide treated nets [ITNs], maternal and child mortality). - Identification of and dialogue on areas needing improvement and planning action to improve. - Advocacy issues to be taken up to the next level. - Recognition of the CHW of the month, based on data. | CHC meetings and the procedures guided by this implementation guide This was an update to the Community Health Strategy | Evolving policy content |
| **2.1.3 Level 2 Management Committee:** This committee should have 12 members with equal representation of the community units served. The chair and treasurer should be elected from among members, while the secretary should be the facility in-charge.  The CHEW should be included and eight other members appointed by  CHCs. The election of the level 2 committee chair and treasurer should be supervised by the DHMT, and they should come from the different community units. Each CHC will nominate amongst themselves up to five people to serve on the level 2 committee. | The implementation guide provided for a Level 2 management committee that would comprise CHC members from different community units | Evolving policy content |
| **2.1.4 Level 3 Health Facility Management Committee**  The committee should have 14 members representing level 2 units served within the catchment area. The level 3 facility in-charge and the Public Health Officer (PHO) in the division will be *ex officio* members (that is, members by reason of their position). The chair and treasurer will be popularly elected, while the level 3 facility in-charge will serve as the secretary. Election of the 14 level 3 committee members should be held at the District Officer’s (DO) baraza supported by representation from the District Health Management Team (DHMT). There should be 2–3 members from each level 2 committee in the catchment area, paying | Another level of health facility committee to discuss CHS matters Original strategy sought to integrate community health strategy in decentralized structures at District level | Evolving policy content |
| **Launching the community health strategy**  STEP 1: Creating Awareness  STEP 2: Situation Analysis  and Household Registration  STEP 3: Planning Actions for Improving Health Status | Process of establishing community units does not involve selection of CHCs. CHCs had no role in establishment of CHCs and selection of CHWs | Evolving policy content |
| **4.1.3 The CHC** A community health committee (CHC) is a group of people who are charged with the responsibility of leading community health action at the community unit level. The committee is composed of 8–12 people selected from the community. Membership must be sensitive to gender balance and equal representation of villages and all interest groups in the community.  The CHC elects officials from among the members: Chair (a respected community member), secretary (the CHEW) and treasurer (a CHW). The CHC has regular meetings that relate to the 100-day improvement cycle as well as community dialogue and action days. When they meet they discuss health-related community issues and review progress of households, CHWs and CHEWs on the basis of planned action for health guided by available data. Village and activity specific data are presented for dialogue and planning to ensure adequate targeting of areas and specific interventions. | CHC defined as a group of people charged with responsibility of leading community health action | Evolving policy content |
| The selection of members to the CHC is led out by the administrative head of the community unit, an Assistant Chief.  The respective level 3 management committee facilitates the process by sending representatives to attend meetings organized by the administrator for the purpose of selecting CHC members.  The characteristics of people to be identified are explained and then  consenting nominees are identified for consideration by the *baraza*, with attention to inclusive representation as described in Chapter 3. The following characteristics are considered in the selection:   - Residency in the area. - Ability to read and write. - Demonstrated leadership qualities. - Demonstrated role model in positive health practices. - Representative of a constituency in the community (village, faith communities, youth, disabled, women, etc.). | Selection process was vaguely described. The guidelines and Community Health Strategy had clear training content for CHEWs and CHVs but none for CHC | Evolving policy content |
| **Setting up the CBHIS**  The system collects information to help the CHC plan and manage health activities at the community level. Within parameters set by MOH, each CHC should decide on the scope of their CBHIS, guided by the CHEW.  On the basis of their experience and available information from the community, the CHC prioritizes the problems that determine the indicators to be included.  Figure 7.1 outlines how an information system is established. After prioritization and agreement on possible courses of action, the CHC with the support of the CHEW plans specific actions to improve the community health situation. To monitor and evaluate the actions and the level of improvement achieved, the CHC identifies the type of information to be collected, who collects it and what tools are necessary. In addition, the committee has to describe how the information will be collected, analysed, disseminated, utilized and stored for future use. | - Original guidance expected CHCs to participate in defining the data system that will help in decision making. - No clear guidance and training contents on how to set up the CBHIS and run it. - No indicators for CHCs data collection was more focused on service delivery activities - CHBIS formats not clearly defined. - Training on CBHIS not explicit | Evolving policy content |
| 1. **[Urban] County Community Health Services Act 2019** | | |
| CHCs shall be in charge of more than one community unit  Composition of CHC   - 7-13 members elected by community members under supervision by Chief / Ward Administrator - CHA shall be advisor / Secretary to CHC - CHV shall be treasurer - Not more than two thirds should be from one gender - CHC should represent youth and marginalized persons - Membership limited to a maximum of two 3 year terms | The county level policy contextualized the implementation the community health strategy so that CHCs could oversee more than one community unit, especially since community health services in urban settings are implemented in urban informal settlements that have high population density | Legal framework that addresses implementation of CHC policy |
| Roles of the CHC   - CHC shall hold at least two public forums to share information on activities of the CHC and seek input from the public - Foster community development that encourages public to actively participate in health planning and service delivery - Develop community profile (demographic, epidemiological, health practices etc) - Prepare and maintain an inventory of community based health services - Assessing community health needs - Develop community health plan | The county level policy on CHCs provides specific guidance on the roles of CHCs. These roles have been modified to suit urban settings where community health services are offered | Legal framework that addresses implementation of CHC policy |
| **Financial provisions**   - Community Health Service funds shall be appropriated by the County Assembly in each financial year - Monies received from national government (conditional and non-conditional grants) - Grants and donations from donors | The county level policy specified how the community health services will be sustained through budgetary allocations. It will be important to review how this budgetary allocations will be sustained in the future | Legal framework that addresses implementation of CHC policy |

**REFERENCES**

1. Dalglish SL, Khalid H, McMahon SA. Document analysis in health policy research: the READ approach. Health policy and planning. 2020;35(10):1424-31.
